# A narrative review of health research capacity strengthening in low and middle-income countries: lessons for conflict-affected areas

**DOI:** 10.1186/s12992-019-0465-y

**Published:** 2019-03-26

**Authors:** Gemma Bowsher, Andreas Papamichail, Nassim El Achi, Abdulkarim Ekzayez, Bayard Roberts, Richard Sullivan, Preeti Patel

**Affiliations:** 1 Conflict and Health Research Group, Department of War Studies, King’s College London, London, UK; 2 R4HC-MENA, Department of War Studies, King’s College London, London, UK; 3 R4HC-MENA, Global Health Institute, American University of Beirut, Beirut, Lebanon; 4 RECAP, Department of Health Services Research and Policy, London School of Hygiene and Tropical Medicine, London, UK; 5 R4HC-MENA, Institute of Cancer Policy, Conflict and Health Research Group, King’s College London, London, UK

**Keywords:** Research capacity, Health research, Conflict, LMIC, Global health

## Abstract

Conducting health research in conflict-affected areas and other complex environments is difficult, yet vital. However, the capacity to undertake such research is often limited and with little translation into practice, particularly in poorer countries. There is therefore a need to strengthen health research capacity in conflict-affected countries and regions.

In this narrative review, we draw together evidence from low and middle-income countries to highlight challenges to research capacity strengthening in conflict, as well as examples of good practice. We find that authorship trends in health research indicate global imbalances in research capacity, with implications for the type and priorities of research produced, equity within epistemic communities and the development of sustainable research capacity in low and middle-income countries. Yet, there is little evidence on what constitutes effective health research capacity strengthening in conflict-affected areas. There is more evidence on health research capacity strengthening in general, from which several key enablers emerge: adequate and sustained financing; effective stewardship and equitable research partnerships; mentorship of researchers of all levels; and effective linkages of research to policy and practice.

Strengthening health research capacity in conflict-affected areas needs to occur at multiple levels to ensure sustainability and equity. Capacity strengthening interventions need to take into consideration the dynamics of conflict, power dynamics within research collaborations, the potential impact of technology, and the wider political environment in which they take place.

## Background

Capacity strengthening for health research is a central concern for development initiatives undertaken in Low and Middle-Income Countries (LMICs). For example, the United Kingdom’s Department for International Development (DFID) has since 2006 established health research capacity improvement as one of its key priorities, investing 8–12% of its research budget on relevant programmes [1]. Despite increasing levels of investment in health research capacity strengthening, there is little consensus on how best to design and evaluate programmes targeting the development of capacity in global health, which has led to an evidence-lite and fragmented field of practice. Some research groups have started to address these concerns by outlining potential indicators and processes for enacting replicable research capacity strengthening, however these have yet to garner system wide acceptance within the capacity strengthening community [2].

At present, health research capacity is disproportionately located in the global North; a recent analysis of authorship trends in *The Lancet Global Health* established that only 35% of authors are from and work within LMICs, whilst 92% of articles address interventions in these countries [3]. This imbalance is rooted in historical inequalities and colonial exploitation and replicated by persisting macro-economic inequalities, attendant problems such as the draining of expertise and dependence on funding from the global North, and power imbalances between researchers and institutions from the North and the South [4]. As such, current capacity for conducting health research in many LMIC countries remains limited and undermines the transformation of health systems into sustainable entities within global communities of scientific knowledge generation able to address both global and local concerns [2]. These geographical inequities in health research capacity distribution illuminate wider challenges in the current landscape of research attribution. The low authorship rates of LMIC authors are indicative of a dearth of local training capability and capacity for endogenous researchers. In addition, rates are likely lower than reported since several phenomena such as “token” authorship, double affiliations and “safari research” (i.e., instrumental inclusion of LMIC authors in order to solicit funding or publication favour) are likely to be skewing the current representation of authorship [5].

Research emerging from countries in conflict is even less common despite the dire health needs of conflict-affected populations [6]. Most research in conflict-affected areas is conducted by non-governmental organisations such as Médecins sans Frontières (MSF), given that they have access to such areas that academics rarely have [7]. The focus within such organisations is often limited to operational research so as not to detract from humanitarian aid delivery given financial and human resource capacity constraints [7, 8]. Furthermore, obstacles such as political pressures, instability, undervaluing research, and ethical challenges all compromise the quality and quantity of the work produced, as well as its impact on policy [7–9]. Most conflict-affected countries have received very little attention in the international literature on health research and are overlooked by large funders, who feel that they can only invest where there is sufficient existing capacity to absorb resources and where the risks to conducting research are minimal [10]. Access constraints, weak local research capacity, collaboration challenges and lack of trust in the research process have been previously identified as the key factors that present unique, specific and sizeable challenges to conducting high quality research in conflict-affected regions [11].

Thus, health research in Low and Middle-Income Countries (LMICs) affected by armed conflict is often fragmented, under-developed, or driven by researchers and research agendas from the global North. This has bearing on the type of research that is produced, the priorities reflected in the research, equity within global epistemic communities, and the longer-term development of sustainable research capacity within LMICs. Strengthening research communities in those settings most vulnerable to the population health consequences of epidemiologic, environmental and conflict dynamics is necessary to build the evidence base to address these challenges, and an important remedy for longstanding legacies of inequitable resource and skills distribution.

The aim of this review is to examine the current literature on health research capacity strengthening (sometimes referred to as research capacity building) in LMICs in order to support the undertaking of future capacity strengthening programmes in conflict settings. We have chosen to call this ‘research capacity strengthening’ rather than ‘research capacity building’, since ‘building’ suggests that capacity is completely non-existent. However, in our literature search we have made sure to capture both uses. We understand ‘capacity’ to be about more than specific research skills, but to include the ability to identify and define problems of local and global concern, to prioritise objectives, to form sustainable institutions to achieve these [12], while accessing global epistemic communities on an equal footing. The review’s key objectives are: 1) to determine the levels (individual, organisational, institutional) at which health research capacity strengthening activities have been aimed; 2) to explore key factors influencing successful health research capacity strengthening programmes; 3) to assess how these factors might apply in conflict settings; and 4) to outline priorities for future research capacity strengthening programmes. Key findings from the review are outlined in Table 1.

**Table 1:** Key findings from the review

Key Findings	
● Research capacity strengthening for health in areas of ongoing armed conflict is almost non-existent	
● Most health research in conflict-affected areas is conducted by non-governmental organisations given that they have access to such areas that academics rarely have	
● Authorship trends reflect global imbalances in research capacity and attribution, with implications for the type of research produced, priorities it reflects, equity within epistemic communities, and the development of sustainable research capacity in LMICs	
● Embedding health research capacity across the individual, organisational and institutional levels requires increasing investment and political will nationally and internationally.	
● Future research on – and indeed interventions aimed at – research capacity strengthening in conflict-affected areas should focus on the impact of: conflict itself, power dynamics within research collaborations (e.g. gender, North-South), technology, and the wider political environment, across all three levels (individual, organisational and institutional).	

## Methods

A narrative review of health research capacity strengthening/building literature was carried out. The narrative review methodology (specifically that of a hermeneutic review) was chosen instead of a systematic review methodology, with the understanding that the topic of research capacity strengthening in conflict is one that requires incorporating a diverse range of sources and knowledge-bases, and interpreting these based on judgment and expertise in order to offer insight into a complex and multi-faceted process [13–15]. Furthermore, as the aim of this piece is to draw lessons for capacity strengthening in conflict-affected LMICs (on which there is little evidence) from the evidence base for capacity strengthening in LMICs *in general*, an ‘interpretive and discursive synthesis’ through the means of a narrative review was deemed most appropriate [13].

To find literature for the review, peer reviewed articles published from 1990 onwards were searched by GB between April and September 2018 using two main databases; Ovid Global Health, and Ovid Medline. Keywords used in the review were; health* research*, capacity building*, capacity strengthening*, course delivery*, capabilities*, conflict*, post-conflict* and LMICs. Searches were completed using individual keywords and further filtered by two criteria; English language literature and combinations of keywords. Abstracts were screened first using broad inclusion criteria (i.e. relevance to health research capacity strengthening in LMICs); studies meriting inclusion at this stage were then read in full before the final determination of relevance was made. Both qualitative and quantitative studies were included.

Subsequently, a search for grey literature was conducted on the World Health Organization (WHO) Global Health Observatory (GHO), Reliefweb and the Social Science Relief Network. Further sources, found and/or recommended by AP, NEA, AE, and PP, and by experts on the theoretical background to health research capacity strengthening (such as the Capacity Research Unit at the Liverpool School of Tropical Medicine), were also included. A final search was undertaken using Google Scholar to assess for any key omissions during the search process. Studies reviewed were published between 1992 and 2018.

Articles were mainly included if they described health research capacity strengthening interventions in conflict, post-conflict, or LMIC settings (See Table 2 for definitions of settings). Interventions were included whether they were delivered by domestic organisations, North-South partnerships or international non-governmental organisations (NGOs), research consortia and private philanthropic organisations. Health research capacity strengthening was interpreted broadly to include general skills development programmes, as well as subject specific initiatives for targeting established research gaps. Interventions constitute any initiative delivered at the individual, organisational and institutional level with the overall goal of increasing the capacity for health research in these settings. Further studies were eligible if they encompassed the stated geographic contexts and described health research skills gaps, or challenges and opportunities for research capacity strengthening whether related to specific interventions or not.

**Table 2:** Definitions of types of settings

***Conflict and conflict-affected:*** Conflict, as used here, refers to violent armed struggle between hostile groups, resulting in over 25 battle-related deaths per year [16]. We use conflict-affected to indicate areas that may not be bearing the brunt of violence, but still experience social and political upheaval as a result of conflict, e.g. in the form of an influx of refugees or internally displaced populations.	
***Post-conflict:*** Post-conflict is highly difficult to conceptualise and may refer to the period following a formal surrender, negotiated end of hostilities, or peace talks. It is a period with increased security and peace, although there may be violence and insecurity in certain regions; political and economic reforms and the influx of large-scale private investment and development aid. Some countries are described as post-conflict for up to two decades or more after the end of hostilities; however, this tends to be very context-specific depending on the typology of conflict. Post-conflict peace is typically fragile: nearly half of all civil wars are due to post-conflict relapses [17, 18].	
***LMIC (Low and Middle-Income Country):*** According to the World Bank’s definitions, drawing on 2017 figures, low-income economies have a gross national income (GNI) per capita of $995 or less; the GNI per capita of lower middle-income is between $996 and $3895; and upper middle-income economies have a GNI per capita of between $3896 and $12,055 [19].	

Articles were analysed by GB using the DFID framework [1] for three levels of intervention of research capacity strengthening, which occur at the individual, organisational and institutional levels. Analysis of the studies determined the distribution of interventions across this framework. Further analysis examined all articles for descriptions of the facilitators and challenges encountered by health research capacity strengthening programmes using a grounded coding methodology. Themes from the papers were manually categorised in two cycles: the first cycle used open coding to generate categories and themes; the second cycle used focused coding to confirm, consolidate, and re-organise categories based on conceptual similarity. AP contributed to further consolidation of themes. Hence, the themes used emerged through the review, while the domains that capture the themes are based on an adaptation and refinement of those used by Pang et al. [20], as discussed in more detail below.

This paper forms part of the Research for Health in Conflict in the Middle East and North Africa (R4HC-MENA) [21] project and – in the interest of validation – drafts were shared with a number of colleagues at the American University of Beirut. Findings were also presented at the executive board meeting of the project in Ankara in December 2018, where colleagues from Lebanon, Jordan, the Occupied Palestinian Territories and Turkey were present, and subsequently verified with research partners in the RECAP project which also focuses on humanitarian health research capacity building in conflict-affected areas [22].

### Findings

Returned searches indicated 281 papers eligible for review; after further screening 74 results merited inclusion. Of these, 43 studies detailed specific interventions, while the remaining publications dealt with research capacity strengthening more generally. Three studies (7% of interventions) explicitly examined post-conflict settings in Somalia, Somaliland and Liberia; none examined a zone of ongoing conflict. Over half (53%) of the described interventions take place in Sub-Saharan Africa and 14% in South Asia. Four studies (9% of interventions) engage with health research capacity in the Middle East and North Africa (MENA) region, encompassing the United Arab Emirates, Qatar, Iran and Turkey. Only one study examines an intervention in the North African sub-region – specifically Tunisia, Algeria and Morocco. Two studies (4.5%) examined South American interventions, specifically in Brazil and Latin America and its diaspora.

### Levels of intervention

The UK’s DFID [1] have outlined three levels of intervention at which research capacity strengthening activities occur; the individual, organisational and institutional level. The individual level primarily includes the delivery of workshops, online teaching, and personal mentorship for the development of skills in selected researchers. The organisational level is the level at which the university or NGO operates and includes such activities as funding system development, curriculum development and research process development, such as Institutional Review Boards (IRBs) and ethics committees. The institutional level refers to broader dynamics that influence the research context, such as the regulatory context, incentive structures and political motivation towards research resource base development (Table 3).

**Table 3:** Studies Addressing the DFID Research Capacity Levels of Intervention

Level	Intervention Aims^a^	Studies
Individual *n* = 24	Strengthening individual capacities through: ■ Mentorship of researchers ■ Research methodology workshops ■ Policy and influence training	[38, 41–45, 56, 65–81]
Organisational *n* = 11	Improving organisational structures, processes and procedures related to research through: ■ Developing capacity for research programme coordination, grant applications, teaching delivery in universities, think tanks, NGOs, etc. ■ Funding system development ■ IRB system development ■ Curriculum development	[23, 31, 82–90]
Institutional *n* = 8	Creating an environment where research can be conducted by setting political, economic, and technical standards and regulations, by addressing: ■ ‘Rules of the game’ ■ Incentive structures ■ Political and regulatory context ■ Resource base development	[23, 24, 27, 28, 91–94]

^a^Adapted from DFID [1]

The majority of efforts have been directed towards the building of individual skills in health professionals – individual training/workshops and seminar interventions examined in this review account for 56% of programmes (Table 3). It is assumed that capacity strengthening at one level leads to increased capacity at others; however this notion is increasingly under contestation, and the literature suggests that a systems approach that cuts across the levels can produce greater capacity dividends [23–26]. The International AIDS Vaccine Initiative (IAVI) represents an important programme in this respect, given its system-wide approach. As part of a global consortium targeting vaccine development, research capacity strengthening in Africa was a specific goal and associated initiatives were integrated across the domains of scientific skills and training, research infrastructure, community engagement and advocacy [27]. Successfully ascending the DFID framework from the individual to the institutional levels requires increasing investment for system development, and the existence of political will within organisations and across national and international domains.

Institutional intervention necessarily demands high levels of political engagement in the process of capacity strengthening and research prioritisation, but such concerted coordination is both resource intensive and demanding of professionals and key stakeholders. The need to convene around shared goals requires a stable and robust political system able to assert its own objectives without imposing undue influence on research organisations. One such example is the Thai public health community’s efforts at strengthening capacity for tobacco control research, which is unusual globally for its comprehensive approach including taxation and corporate regulatory reforms [28]. In Thailand a tobacco control research community has been built during three phases; 1) discovery of the value of research; 2) development of capacity strengthening processes alongside research governance systems; and 3) undertaking of locally determined research responding to local needs [28]. Essential to this process has been “buy in” from the Thai government and external donors in order to adapt foreign research to domestic requirements and to support greater regional collaboration to build research networks and address the influence and power of the tobacco industry [28]. Clearly initiatives such as this require the mobilisation of a great number of scarce resources, and the challenges inherent in producing what Shiffman and Smith [29] describe as ‘issue attention’ require careful management when establishing a more system-wide intervention. For this reason, it is not surprising that only 19% (Table 3) of reviewed interventions occur at the institutional level.

### Factors influencing research capacity strengthening

The identification of core practices that support the establishment of strong health research systems is an important area for interventions. A strong health research system should comprise of systems and processes that synthesise interventions informed by broader set of determining principles. Pang et al. [20] have argued that strong financing, production and utilisation of research, resources and stewardship are four cardinal features of a well-functioning sustainable health research system. Within these domains can be seen a great variety of practice across health research systems. To better capture the broad range of influences determined from the literature reviewed here, Table 4 breaks their features down into more specific categories that emerged from our thematic analysis. These categories, and the positive and negative influences within them, may guide further undertakings in capacity strengthening initiatives.

**Table 4:** Influencing Factors on Health Research Capacity Strengthening

Domain	Positive Influences		Negative Influences	
Financing & Sustainability	Access to Funding	[28, 32, 38, 95]	Inability to access funding	[25, 31, 38, 89, 94, 96–99]
	Continuity of funding	[28, 30, 31, 38–40, 89, 97]	Short-term research funding	[30, 99, 100]
Resources	Adequate and appropriate infrastructure	[23, 28, 30, 31, 38, 39, 46, 74, 94, 95, 99, 101, 102]	Demands of clinical service delivery limiting staff participation in research	[38, 39, 89, 92, 96, 103]
Stewardship & Leadership	North-South Partnerships	[24–26, 28, 34, 39, 46, 88, 91–94, 102, 104–106]	Weak scientific leadership	[39, 40, 92, 96, 99, 102]
	Capable leadership	[23, 30, 39, 71, 95]	Integrating new initiatives into existing systems	[25, 107]
			Strong external (political) influence on institutions	[39, 72, 94, 108]
Mentorship	Sustained mentorship	[24, 25, 30, 38–40, 44, 73, 89, 101, 105, 109]	Absent mentorship	[76, 96, 99]
Partnerships	Creating networking opportunities	[25, 94, 110–112]	Differing expectations of partners	[33]
	Equity in collaboration/shared decision-making	[2, 3, 23, 26, 30, 33, 37, 39, 45, 88, 106, 111, 113–115]		
	Sustained collaboration over time	[114]		
	History of collaboration/pre-existing relationships	[23, 33, 114]		
Production & Utilisation of Research	Ability to attract young dedicated scientists	[24, 39]	Poor incentives to conduct research	[24, 39, 91, 116, 117]
	Research addressing policy gaps and local needs	[23, 24, 28, 30, 46, 92, 93, 102, 104, 114]	Culture/attitude barriers	[38, 117, 118]
	Local leadership and claim-making	[23, 26, 28, 45, 46, 69, 88, 93, 102, 114]	Difficulty publishing in international journals/scarcity of local journals	[38, 44, 119]
	Research governance structures	[23, 28, 30, 89, 91–93, 114, 120]	Low staff and stakeholder retention	[25, 30, 102, 108]
	Favourable political conditions	[24, 28, 30, 39, 117]	Neglect of skills	[25, 99]
			Failure to link research to policy	[26, 102, 116, 121]

Financing is regularly cited as the critical factor limiting the development of health research systems in LMICs, as indicated in Table 4. Access to sufficient, sustainable and long-term funding was a key determinant of research capacity strengthening success. Yet, the ability to access financial resources is curtailed by several factors, such as levels of public expenditure on research and the disbursing structures of funding bodies. For example, the review by Ismail et al. [30] of health research in the Eastern Mediterranean region details evidence that this region counts among the lowest investors globally in research and development activities, averaging around 0.3% of gross domestic product (GDP) compared to 1.8% in the UK, and 2.8% in Japan. Meanwhile, the ability of LMIC researchers and research groups to access financial resources from international funding bodies and donors is hampered by asymmetries in how grants are allocated, requirements for partnerships with Northern institutions, and the disbursement of funds *within* such partnerships [31–33].

Relatedly, stewardship emerged as a further influencing factor on health research capacity strengthening. North-South partnerships, in particular, offer a means to consolidate the benefits of knowledge and resource transfer at the individual level, whilst capitalizing on organizational learning in order to generate onward systemic and procedural benefits [34]. There are several examples of such international collaborations such as the Task force on Malaria Research Capability Strengthening coordinated by the WHO, which disburses grants to African research groups to work in partnership with US and European groups, as well as facilitating networking and educational activities for graduate and postdoctoral researchers [34]. The US National Institutes for Health has also promoted stewardship in research capacity by linking US institutions with leading research centres in LMICs such as India, Mali and Uganda [35]. Leading initiatives from the UK include the £1.5 billion Global Challenges Research Fund that explicitly addresses the development needs of overseas development assistance (ODA) recipient countries; the Wellcome Developing Excellence in Leadership, Training and Science Initiative (DELTAS) for research and training programmes led by African scholars; and the £735 million Newton Fund, which includes partner countries in both decision-making and financial contributions. These innovative models need to be studied – major funding schemes from resource-rich settings should be designed to encourage and leverage local LMIC co-funding that leads to better ownership and sustainability of research programmes [36].

A caveat to the benefit of North-South collaboration is that to be effective, these collaborations need to be equitable. The way partnerships are established, whether there is clarity and alignment on expectations, how funds are managed and by whom, how research priorities are identified, and how benefits for both sides are distributed *and* perceived can all have bearing on both the equitability and success of research partnerships [26, 33, 37]. We have included ‘partnerships’ as a stand-alone category in the table above to reflect the importance of this domain.

Mentorship is a critical interpersonal theme and is a recurrent element of effective programmatic work in health research capacity strengthening [25, 30, 38–40]. Mentorship can take place between students of health research and their teachers; a lack of effective doctoral supervision has been cited as a barrier to the development of broader national health research systems [40]. Equally the importance of international linkages with researchers in other institutions with more established research cultures, has been found to be a beneficial form of mentoring for East African clinical research trainees, and is thought to enhance the quality of research output [38]. Mentorship is also an important dynamic as programmes are deployed on an organisational basis, facilitating the transfer of knowledge between groups delivering training programmes and their Northern or Southern partners [25, 30, 39].

Failures to link existing health funding to the production and utilization of research has resulted in strikingly low publication outputs in various LMIC settings [30]. The most effective programmes at generating rapid increases in research output are interventions delivering thematically focused operational research training programmes such as the SORT-IT model. MSF, Partners in Health, the International Union against Tuberculosis and Lung Disease, and the American Thoracic Society have all reported successful programmes delivering multi-national operational research programmes in LMIC settings, with clearly linked research outputs including publications numbering in the 1000s over a combined 20-year period [41–44]. Delivery of these programmes has been facilitated by the fact that they are spearheaded by organisations with secure funding sources with the means to bypass the difficulties of working within national systems.

However, at times these programmes are found lacking in their scope for scale-up and integration into sustainable long-term national and regional research governance systems [30]. A number of important issues arise in relation to this finding including limited institutional and individual financial incentives for conducting research, political sensitivity towards findings, and a poor connection of research to policy activities [30]. Key to sustainable capacity strengthening in this domain is a shift in ethos towards strong local involvement in leadership, policy-making and priority setting [26, 45, 46]. Such an ethos requires reflexivity in programmatic design, coupled with a shift from focus on end outcomes measured only as publication outputs and number of training workshops, to a greater emphasis on quality, sustainability and utility of research [10, 24]. One potential approach to a better linkage between the stewardship, financing and production and utilisation of research domains is that of ‘embedded research’ which brings together researchers, implementers and policy-makers to set research priorities *and* conduct the research as a way of bridging the gap between knowledge production, policy uptake and implementation [47, 48].

### Health Research capacity in conflict

Despite growing global interest in health system strengthening in conflict, there is very limited specific literature on health research capacity strengthening in this context [11, 49]. Of the studies identified for this review, none discussed research capacity strengthening interventions in an active conflict zone. Three took place in conflict-affected areas (Somalia, Somaliland & Liberia), all of which are now in various phases of post-conflict reconstruction. In these environments all the usual challenges of capacity strengthening processes are present, however the conditions of political precarity, resource scarcity and instability are intensified [50]. So too are the healthcare demands on limited services, accentuating the gulf between research needs and gaps. Study populations can be difficult to reach, and building sustainable partnerships that recruit and retain research staff can be a challenge [51]. Previous reviews have established that capacity strengthening efforts have focused on settings with at least some existing capacity rather than those where it is almost entirely lacking [10].

This is not to say that capacity strengthening efforts are not taking place, but rather that they are ad hoc and/or understudied. Examples of such efforts include responses to the Syrian conflict and the influx of Syrian refugees to Lebanon by academic institutions like the American University of Beirut [52], which have been engaged in redesigning and delivering modules and trainings to address the extant health situation. Moreover, there are ongoing partnership projects between the North and South like the Research for Health in Conflict in the Middle East and North Africa R4HC-MENA [21] and RECAP [22] projects that this study forms part of, that aim to support preparedness and response to conflict by strengthening research capacities. Since these projects have started relatively recently, research outputs are forthcoming. Meanwhile, NGOs at the frontline in humanitarian settings are generally more concerned with implementation rather than knowledge production, and much of their work and experience remains understudied, or at best is found in grey literature [8, 53, 54]. As a result, the literature is sparse.

In part, this is because the challenges of conducting research in conflict environments have militated towards the delivery of training programmes via local actors with limited stewardship, high financial and resource costs and weak research capacity strengthening [50]. The sparse literature does indicate certain key influencing factors and tactics for successful research capacity strengthening in conflict-affected areas. For example, as in research capacity strengthening for health more generally, the importance of thinking beyond the individual level and adopting a systems approach is also emphasised by a report for Elrha – which provides funding for improving humanitarian outcomes through partnership, research and innovation [55] – on research for health in humanitarian crises [51]. Of course, system-wide approaches can be very challenging in many conflict-affected countries and environments given political constraints and the existence of conflict, but might be possible in regional hubs such as Lebanon, Jordan, Turkey, Brazil, and Kenya, for example.

Furthermore, novel approaches such as web-based learning via online platforms as a means of crossing geographical and political boundaries have emerged as potential modes of knowledge transmission and evidence accumulation [56, 57]. The adoption of technologies for research in conflict has been proposed as a potential tool for teaching research skills in the Palestinian territories as a means of overcoming the barriers imposed by professional groups being separated by checkpoints and bureaucratic delays [58]. Local ownership has again been identified as an essential priority; a longstanding initiative between the Swedish Agency for Research Cooperation with Developing Countries and the Somali Academy of Science and Art (SOMAC) has emphasised the importance of long-term locally directed programmes supported by well-resourced international partners in the aftermath of conflict and during the process of rebuilding during ongoing fragility [45]. Work is also being undertaken to examine how to best encourage translation of research in humanitarian crises into the policies and practice of humanitarian organisations by linking research, policy-making, and humanitarian communities together, for example by the Advancing Health Research in Humanitarian Crises project of the Fogarty Center for Global Health Studies [59].

## Discussion: implications for research capacity strengthening in conflict-affected areas

Despite increasing funding, the field of health research capacity strengthening in conflict remains empirically and conceptually under-developed. Interventions appear to dedicate attention to concepts on an ad hoc basis, and areas of neglect emerge according to persisting patterns of inequity and practical limitation within current systems. Table 4 proposes key influences that programme developers and local actors may adopt as they design interventions. Of these influences, leadership and mentorship, North-South partnerships, adequate resources and access to funding emerge as priority areas. The importance of international collaboration emerges as a crucial means of developing capacity throughout these domains. Research consortia spanning regions and a range of institutions and practitioners may offer the clearest means with which to mobilise funding, facilitate mentoring and engender political favour around priority issues.

Of course, these influences depend to a great extent on context, and in conflict-affected areas the context will be highly determinative of research capacity strengthening success. We suggest six areas that warrant particular attention in the context of conflict. The first is a call for further research and exploration of conflict and its clearly deleterious effects across the individual, organizational and institutional tiers of intervention. Such explorations are necessary in order to trace conflict specific trends, as well as for the generation of a research base to inform policy and regulatory responses to conflict research needs as they arise. Relatedly, we note a lack of quantitative studies into health research capacity strengthening in general, and that surveys on individual research capacity strengthening needs and intervention studies assessing the effectiveness of capacity strengthening activities (e.g. improved individual knowledge, skills and practice) ought to be conducted, including in research in conflict-affected areas.

Second, instability has naturally tended to motivate the delivery of short-term programmes with limited integration into residual governance structures and consideration for sustainability, particularly of financial resources. Examining the role of large consortia able to mobilise resources and preserve processes to be embedded during post-conflict recovery may offer potential for the challenging prospect of research sustainability in these settings. A more radical solution for lacking financial resources would be to look towards collectivising research funding in regions most acutely lacking health research skills. The WHO’s 2012 Report on *Research and Development to Meet Health Needs in Developing Countries: Strengthening Global Financing and Coordination* argues that taxation in some form has the greatest potential to address the dearth of funding sources available in LMICs. The conclusion of this report asserts that all nation states should commit to spending a minimum of 0.01% of GDP on government funded research and development, and that developing countries with potential research capacity should aim to dedicate 0.05–0.1% to such activities [60]. A recent Lancet commentary reasserted the call for taxation on large-scale private industries such as mining to fill this capital gap [46]. This approach could serve as a more equitable taxation system and serve to redress specific morbidity and mortality burdens brought about by widespread industrial activities such as mining, chemical processing, and agribusiness [61].

The third necessary intervention is an examination of gender as an important locus of inequity in health capacity research, and in health systems more generally [62] . The inclusion of female professionals in capacity strengthening programmes should be seen as essential. However, the inclusion of gender analysis across all domains including financing, policy, community engagement and advocacy should be a more ambitious target [63]. There is a necessary requirement for a more structured approach to include gender as a concern at all levels of intervention, including, or perhaps especially, in conflict [64].

The fourth domain also relates to inequity and power distributions within research capacity strengthening projects, namely the location of decision-making power within North-South partnerships. Dean et al. [33] note that while there has been a proliferation of frameworks and principles to guide effective research capacity strengthening projects, few of these are developed from the perspective of LMIC researchers (of all levels, not just lead researchers). Nor is there much existing research that looks at research partnerships from a southern perspective or contrasting North-South to South-South partnerships [37]. This is a dual concern for equity and for success and effectiveness; involvement of partners on an equal footing is likely to engender sustained collaboration and building of networks.

A fifth conceptual domain is technology and its potential for facilitating capacity strengthening. The literature raises a number of opportunities for the productive introduction of technology to assist mentorship, networking, project management, and equitable North-South partnerships [25, 39, 57]. Technology’s usages have been emphasised specifically in the settings of conflict and fragility for its ability to allow professionals to bypass geographic and political boundaries whilst accessing high-level instruction. However, this comes with a requirement for the existence of adequate infrastructure which can be challenging in conflict-affected areas.

The final domain is the issue of politics as an important international and national process determining the landscape of health research capacity strengthening, particularly if capacity is to be developed beyond the individual level. Societal and political cohesion is a clearly established influence on capacity strengthening [39], and mobilising the resources needed for a health research system requires issue attention amongst an array of actors as part of a concerted effort at health system strengthening [29]. Clearly the combination of fragile political systems with overlapping conflict adds even more challenges to the implementation of robust and resilient systems; post-conflict planning should therefore integrate health research capacity strengthening into its health system strengthening agendas.

### Study limitations

The review methodology sought to capture a thorough representation of lessons learnt from health capacity strengthening projects in LMICs and to assess their applicability in conflict-affected areas. In order to ensure that the key themes were captured in sufficient detail the findings of the review were subject to repeated discussion and shared with partners across the research consortium involved in this study. A crucial limitation, as discussed throughout this review is the unbalanced authorship attribution of studies in the field of research capacity strengthening/building. The majority of studies in this review are written by Northern authors and published in English language mostly in academic journals based in the Global North; it is hoped that by contributing to the development of a more equitable health research capacity agenda through specific programme development and broader discussion, this inequity may be remedied.

A second limitation is that health research capacity strengthening is an evolving concept with a variety of definitions. We chose to follow the framework conceptualised by DFID [1], but acknowledge that some of the terms used are contested within the field and that the adoption of this framework may obscure alternative perspectives. In particular, we note the absence of frameworks that take into consideration the specific challenges presented by settings affected by conflict. We therefore argue that there is an urgent need to conceptualise a framework that is relevant to conflict settings.

## Conclusion

This review has made it clear that the evidence base for health research capacity strengthening is limited, particularly in conflict-affected areas. In part, this is because effective research capacity strengthening is both intangible and highly context-specific, and positive outcomes may not materialise immediately, nor be directly attributable to an intervention. Nevertheless, there are certain factors that do seem to positively influence strengthened research capacity: addressing the individual, organisational and institutional level in tandem; adequate and sustainable funding and resources; capable and shared leadership within sustained and equitable partnerships; mentorship; the development of professional networks; and the linking of research to policy and practice, among others. Most of these factors are clearly mutually constitutive, indicating that sustained research capacity strengthening requires the creation of enabling environments within which skilled researchers feel that their research is valued and impactful and they can develop long-standing collaborations with other researchers. Understanding gendered and North-South (as well as other) power dynamics, and the political context in which research capacity is being built, are all important for creating such an enabling environment. Undoubtedly, this is all the more challenging in conflict-affected areas, where stability, leadership, financing, access and connectivity might conceivably all be lacking. There is a need for more research on health research capacity strengthening for health more generally, but particularly on innovative ways of overcoming some of these additional challenges posed by conflict and instability.

## Abbreviations

DFID: Department for International Development
GHO: Global Health Observatory
GNI: Gross National Income
IAVI: International AIDS Vaccine Initiative
IRB: Institutional Review Board
LMIC: Low and Middle-Income Country
MENA: Middle East and North Africa
MSF: Médecins sans Frontières
NGO: Non-governmental organisation
ODA: Overseas Development Assistance
R4HC-MENA: Research for Health in Conflict (MENA)
SOMAC: Somali Academy of Science and Art
WHO: World Health Organization
